# Notes and News

**Published:** 1890-11-29

**Authors:** 


### Notes and News.

Collected and Communicated.

Miss Gethen acknowledges with thanks scrap-books from "E. C. G.," "Miss Sutton," and "Three Children," also a packet of pictures from "H. M." All these will be forwarded to the children in the Colombo Leper Asylum.

The inauguration of the session at the Children's Hospital, Dublin, was held on the 17th inst. Dr. M'Cullagh delivered an address on "The Physical Education of Children," in the course of which he condemned the high pressure system in connection with education. He expressed a hope that in the near future the education of the body should go on side by side with that of the mind. Dr. Grimshaw took the chair, but the attendance was not very large.

The new hospital at Kirkcaldy was gifted to the town by Mr. M. B. Nairn, of Rankeilour, well known in the floorcloth trade. The building, which is on the cottage hospital principle, is situated towards the east of Kirkcaldy, near the old Castle of Ravenscraig, on land purchased by the donor from the late Earl of Rosslyn. It fronts the road leading between Kirkcaldy and Dysart. It is fitted up with light beds, and surrounded by fine grounds. The new hospital is to be used for surgical purposes and acute medical cases. In addition to the building, which cost about £3,000, Mr. Nairn has given a donation of £600 towards its endowment. The hospital was opened on November 15th.

An anxious correspondent wants to know if it is injurious to the health to grow chrysanthemums, and if so, why? Our correspondent writes a good hand, and does not spell badly; what a pity she lacks common sense! No pursuit could be healthier or more conducive to happiness than that of gardening, which includes the growing of chrysanthemums, and it would, indeed, puzzle a medical man to say why this one plant is more injurious to cultivate than others. The spirit of superstition is indeed still rife amongst us! Last week Mr. Justice Cave was told in court, by a witness speaking on oath, that the presence of a ghost in the room was demonstrated by the fact that the stopper of the Yorkshire relish ottle jumped out of its proper place, and fell on the table.

The Royal Albert Hospital, Devonport, treated 478 in-patients last year. Eleven patients have been treated in the pay wards. The Nursing Institution continues to do good work, and altogether the report is most satisfactory. Sir Richard Harrison took the chair at the annual meeting, and there was a good attendance, including Lord St. Levan and Dr. Bickersteth.

The annual meeting of the Zenana Medical College took place on Tuesday at Exeter Hall. Unlike most annual meetings, the proceedings were made bright and interesting, and there was a large attendance of friends. A musical service was first held, and then various speakers explained the work of the college, and what was more thrilling, explained the work the former students of the college are now doing in foreign lands. That women should minister to women is a truth daily becoming more patent, and common sense adds that the ministering woman needs training as much as any man needs training before he can enter on any profession. Hence the worth of the Zenana Medical College, in St. George's Road, S.W., which appeals for support and help.

The Seaside Convalescent Hospital at Seaford, in Sussex, brings out its this year's report in a pretty ornamental cover. As a charity it is an excellent one, and we wish it every success in the future, as it has had in the past. Any of our readers who happen to be staying at Seaford or Newhaven would do well to acquaint themselves with the arrangements and working of the institution. The only fault to be found with the Seaford Hospital is that it is too large to be a "home" in the true sense of the word, and that though it calls itself a hospital it will not take anyone needing medical or surgical treatment. However, it is not large enough for the many applicants who apply for admission during the summer months, and we know well how grateful the patients, lucky enough to be accepted, are for their stay by the sea.

Why is it that well-intentioned people who try to improve the souls or minds of the proletariat on Sundays pay so little need to their bodies? The atmosphere of most places of worship is absolutely foul. Nor are the churches alone to blame in this respect; the ventilation of the Positivist Hall in Fetter Lane, and still more of St. Andrew's Hall, where the National Sunday League holds out, is most defective. To draw the people in out of the fresh night air, and fill their lungs with poisonous fumes, can scarcely be forgiven because you also give them a little intellectual food. It ought to be incumbent on every body that draws together a large congregation to grant that congregation a certain cubic space of air. The practical Salvation Army is the least sinner in this respect; most of their halls and refuges are fairly well ventilated.

Belfast Royal Hospital had a little better news for its supporters at last week's annual meeting. The report of the Board of Management contains the following: "The increase in general subscriptions, church collections, Hospital Saturday collection, but most of all in the collections taken up in mills, factories, and warehouses shows that all classes are recognising the vast amount of work that is done daily in attending to the sick and suffering; and by none is this more fully recognized than by the working classes, who, through the exertions of their own committee (to whom our best thanks are due), have raised a larger amount than in any preceding year, and who have pledged themselves to even greater exertions during the year now commenced." From what was stated by two or three gentlemen present, attention must be given to the important question of enlarging the hospital.

The following vacancies are announced this week, the amount of the salary in each case being given after the name of the institution :—

**Resident Medical Officers.**

The Hospital for Sick Children, Great Ormond Street, House Surgeon—£50, board, &c.
West London Hospital, House Physician and House Surgeon board, &c.
South-Western Fever Hospital, Clinical Assistants.—board, &c.
Colchester Hospital, House Surgeon and Apothecary—£100, board, &c.
Stafford Infirmary, House Surgeon—£100, board, &c.
Bolton Infirmary, Senior House Surgeon—£120, board, &c.

**Non-Resident Medical Officers.**

County of Lanark, Medical Officer—£500.
Sheffield Public Hospital, Physician.
Taunton Hospital, Honorary Dental Surgeon.
St. John's Hospital, Leicester Square, Assistant Physician.
Cancer Hospital, Fulham Road, Dispenser—£100.
London Hospital, Surgical Registrar—£100.

---

Christmas parcels have been received from Mrs. Masters, The Abbey, Cirencester; from Mrs. Fearon, Banstead; and from Miss Coates; and M. A. S., Bexley Heath. We regret delay in acknowledging the last, and thank all our friends who are workingfor the adult patients.

---

Hospitals receive as much support from literary and artistic circles as from the aristocracy. Of course the substantial support of City men stands first, but after that comes the help from professional people. Mrs. Keeley and Mr. Ashby Sterry are very old and true friends to the Hospital for Sick Children, and when Mrs. Keeley entered her 86th year last Saturday, a tiny deputation of invalid children presented her with a bouquet and an address written by Mr. Ashby Sterry. We quote a few lines from the address :—

" How oft at eventide, with lowered light,
When tender nurses watch us through the night;
And sweet-toned whispers strive to soothe our pain,
Oft has the tale been told and told again—
How the Good Fairy came two years ago,
And wrought her subtle spell within our Show;
And how she spoke and pleaded till she drew
Bright tears from eyes—bright gold from pocket, too!
She worked such wondrous charms, she made them bring
So many feathers for our brave New Wing;
She made it grow—'tis now almost complete—
And feathered *such* a nest in Ormond Street!"

---

Croydon Board of Guardians last week had before them two important questions. Dr. Carpenter drew attention to the Master's report, in which it was stated that, in consequence of a number of diphtheria cases being under treatment, he had been obliged to purchase a quantity of chloride, arrowroot, corn flour, and lemons, and that he required two gallons of brandy immediately, and 2lbs. of fresh butter. He had no hesitation whatever in saying that when brandy was administered to diphtheretic patients, the cases were almost certain to end fatally. His own experience was strong against the use of brandy, which, when used, was not only given to the patients, but was freely partaken of by the attendants and nurses, on the supposition that it was a protection against the insidious growth of the mysterious disease. The Board listened to Dr. Carpenter and then proceeded to consider the question of appointing a steward and matron of the Infirmary. It was proposed that the steward should be paid £125, and the matron £100, and that the latter should be a trained nurse. But the Board grew pensive when these sums were mentioned, and finally the motion fell through. Well, if only the Board had the sense to connect these two incidents, they would see that it would be economy to pay well to secure good service.

At the opening of the Hospitals Association meeting on the 20th inst., the Chairman (Sir Andrew Clarke) said he thought it was fitting that the first meeting of the Hospitals Association since the death of Lady Rosebery should pass a vote of sympathy with Lord Rosebery in his bereavement. Lady Rosebery was one of the most loyal friends of nurses in all countries, and her premature death had not only lost to them a warm supporter, but every good work in this country, every good work almost in neighbouring countries, would suffer from it. A most devoted and helpful wife, a true, simple, noble woman, her death was something like a national calamity. Mr. Burdett added that Lady Rosebery president of the Jubilee Nurses in Scotland, a patroness of the National Pension Fund, and one of the most active and helpful of women connected with the Nursing World.

---

The discussion following the paper was interesting, Mrs. Hunter representing the opposition, and declaring the picture drawn by Mr. Burdett to be too favourable. Miss Rosalind Paget spoke in defence of the London Hospital, against which Mrs. Hunter had made an attack, and stated that she had been a worker at the London Hospital for ten years, and therefore spoke with authority. Dr. Gilbert Smith, in the course of an able speech, showed that Mrs. Hunter was wrong as to nurses finding their hours long. If there was a need to reform their hours, how much more need was there to reform the hours of the poor doctor. Nurses' hours were no longer than the hours of labour of the medical staff, or the visiting physician, and certainly not more than those of their chairman. The comparison with the eight hours movement was illfounded as, in the first place, as far as he could find out, such a movement was not in general favour; while in the second they must remember that the working-man was engaged during his day on hard manual labour the whole time, while nurses during their twelve hours' day had a good deal of sitting down, and were not hard at work during the whole time. Mrs. Dacre Craven gave a graphic description of her experiences as a lady probationer five and twenty years ago. Then nurses had tea and bread and butter only for breakfast at 6.30 a.m., a slice of bread and butter (taken away from breakfast) and a glass of milk at 9 a.m. Dinner came at one, and of these she could easily give the *menu*, as on four days in the week they had mutton, and on the other three beef. Tea was taken at five, and supper at nine, the supper consisting of what was left over from dinner. Mr. T. Ryan having explained the diet table of St. Mary's Hospital, of which he is the secretary,

---

Mr. Burdett, in reply, expressed a wish that Mrs. Hunter could enter as a nurse at one of the London hospitals, so that she might become practically acquainted with a subject in which she took so great and public an interest. Votes of thanks to the Treasurer of St. Thomas's Hospital for allowing the use of the Governors' hall for the meeting; and to the Chairman for presiding, were also passed.

---

The Cornelia Hospital, at Poole, of which Lady Wimborne is the bountiful godmother, has just issued a most satisfactory report. This institution first saw the light in the shape of two small cottages; now, one finds it to be a beautiful, airy house, with large rooms, and plenty of nature's grandest remedy, fresh air. Well-manned by a competent crew, this charitable venture is in admirable working order. It has been of immense value, not only to the sailors, who repeatedly incur accidents on the Quay, but also to the general community of working folk, the number of outside patients who come daily for the dressing of wounds, &c., being quite a distinctive feature. We heartily endorse Lady Wimborne's hope that she may count upon active sympathy, on all sides, for the Cornelia Hospital.

